# New York Academy of Medicine—Section on Orthopedic Surgery

**Published:** 1892-04

**Authors:** 


### Society Proceedings.

### NEW YORK ACADEMY OF MEDICINE.—SECTION ON ORTHOPEDIC SURGERY.

*Stated Meeting, February 19, 1892.*

HENRY LING TAYLOR, M. D., Chairman.

Dr. W. R. TOWNSEND presented a girl, fourteen years of age, with rotary lateral curvature. At the age of three years, and after whooping cough, she developed an empyema on the left side, which opened spontaneously. These sinuses continued to discharge for five years, and the three cicatrices, one to the left of the nipple, and two slightly below and to the right, show the points where the openings occurred. When five years old, it was noticed one morning that there was a complete loss of power in the left upper extremity. The mother said that there had never been any curvature of the spine before the attack of paralysis, although the child always slept on the left side, and that the curvature has been steadily increasing since then. The circumference of the chest at the nipples is twenty-four inches, the right side measuring fifteen, and the left nine inches. There is a very marked lateral rotary deviation of the spinal column to the right, extending from the seventh cervical to the tenth dorsal, with compensating curves above and below. There is no torticollis. The breathing space is good, considering the amount of the deformity. The heart is not displaced. There is complete loss of reaction to faradism in the left supra- and infraspinatus, and in the deltoid, and a reversal of the formula with the galvanic current. There is no anesthesia, but marked atrophy of the shoulder and upper left arm. There is a partial loss of reaction in the pectoral, but the biceps, triceps, and forearm muscles react well.

The interesting feature was the relation of the rotary curvature to the empyema and the poliomyelitis. His own opinion was that the empyema probably caused a slight curvature, and that the paralysis had helped to increase it, but that there was no connection between the empyema and the paralysis; in other words, the paralysis was not produced by the scoliosis, but was separate and distinct, and due to a poliomyelitis. He had presented the case chiefly because it was of interest in connection with the first paper announced for the evening.

Dr. Royal Whitman also presented a little girl as an illustration of a pure rotary lateral curvature, caused by anterior poliomyelitis.

Dr. H. W. Berg said that he had had an opportunity of seeing this patient, and had obtained a somewhat different history. According to this version, the patient was still in bed with the empyema, when the family first noticed that she was lying more upon the left side. The occurrence of the paralysis was sudden, and the attending physician allowed her to get out of bed, and at this time the extreme lateral curvature was first noticed. If this curvature were the result of the poliomyelitis, it would not have been so extreme at this early stage, for it takes time for muscles to contract and cause deformity. In this case the paralyzed muscles are on the left side of the body, and the primary curve toward the right, while in cases of lateral curvature due to paralysis, the healthy muscles must necessarily be on the concave side of the deformity. The only way in which poliomyelitis could possibly produce a curvature on the concave side of the deformity, would be in the third stage of this disease, *i. e.*, in the third or fourth year after the paralysis, when the muscles begin to contract into firm fibrous cords.

Dr. Royal Whitman thought if the long supporting muscles were paralyzed, it might be as the previous speaker had said, but in those cases where only the muscles supplying the shoulder were paralyzed, one would expect the curvature to be toward the opposite side.

Dr. Berg replied that the intrinsic muscles are not alone paralyzed in this case. Lateral curvature must follow contraction of the intrinsic muscles of the spine, and not of the long muscles.

Dr. R. H. Sayre had seen a number of cases of lateral curvature dependent upon poliomyelitis with paralysis of the external muscles on the concave side, and hence, he thought, the statement that the convexity is always on the side of the paralyzed muscles could not be accepted without qualification. He had been surprised that German writers took it for granted that empyema curves are not rotary.

Dr. S. Ketch was not prepared to endorse the view that the curvature was mainly due to the empyema ; on the contrary, he thought the patient had that form of curvature usually found as a result of anterior poliomyelitis. Undoubtedly the empyema tended to exaggerate this curvature.

Dr. N. M. Shaffer said that, so far as he knew, the first reported case of lateral curvature due to poliomyelitis had been published in his book in 1876 or 1878. That case had been examined by Dr. Seguin, Dr. Draper, and himself, and they had found the paralysis on the hollow side. On general principles, he believed that Dr. Berg was correct in his statement. In 1881, he had called attention to the fact that a rotary element existed in empyemic curves. It was exceptional for him to find a lateral curvature of the spine, due to empyema, which was not associated with a greater or less degree of rotation. The error probably arose from the fact that Dr. W. J. Little, of London, who first described it, made this mistake, and other writers had perpetuated the error.

Dr. Mary Putnam Jacobi called attention to the monograph by Fulenberg, on lateral curvature of the spine, in which he stated very categorically, that in ordinary typical cases of lateral curvature, the muscles on the concave side are necessarily the stronger, and explains on this principle the mechanism of the production of lateral curvature. His idea is, that it is due to a disturbance in the balance of the muscles of the two sides, whether extrinsic or intrinsic.

Dr. A. B. Judson said that in his earlier studies of lateral curvature, he had adopted, without due verification, the statement of foreign observers that rotation is absent from the curvature caused by pleural disease. At present, he believed that it does not occur, but in a very modified and unimportant degree. The collapse of the chest wall would weaken the action of some of the muscular and fibrous structures which cause rotation by holding the spinous processes nearer the median line than the bodies of the vertebræ. For this reason we may well expect the rotation to be less marked. In the case shown, there is little difference in the diagonal diameters, which is the chief feature of rotation, and is caused, in an ordinary case, by the prominence posteriorly of the right back of the chest, and the complementary prominence anteriorly of the left front of the chest. Here we have prominence, front and back, on the right side, and depression, front and back, on the left side, with but little difference in the diagonal diameters, a condition very unlike the effect of rotation. Still there may be, and probably is, some rotation in the vertebral column of this patient, although its effect on the deformity is not easily recognizable.

Dr. Townsend said that, owing to the fact that in this case one was compelled to rely wholly upon the varying statements of the parents of the child, who were not very close observers, it would be well to be cautious in drawing conclusions from a study of this case alone. He did not agree with Dr. Berg as to the relation of the paralyzed muscles to the concave side.

### VOLUNTARY SUBLUXATION OF THE KNEE PRODUCED BY MUSCULAR ACTION.

Dr. R. H. Sayre showed a child of fourteen months, presenting this condition. The mother first noticed this condition when the child was eight months old. When he was excited, the right knee is pushed in and out with a distinct click. The child was born after a normal labor, and there was no history of injury. He proposed to apply a splint, in order to retain the knee in position.

### AN APPLIANCE FOR THE PREVENTION OF DEFORMITY IN HIP DISEASE.

Dr. Whitman presented a case illustrating this appliance. He believed that the long traction brace was the most useful appliance in these cases, for it assured, as a perineal crutch, a protection which could not be removed by the patient. This was the principal objection to any brace which depended on axillary crutches for its usefulness. Simple fixation of the joint, allowing the patient to walk about on the affected limb, as practised by Thomas and others, did not afford this protection, which he considered the most important element in the treatment of any joint affection. On the other hand, with the simple long traction brace, gradual and increasing flexion of the leg was a very common and troublesome complication. This was the weak point of the brace, and the one most constantly attacked by its opponents. He had therefore attempted to combine the merits of two braces as follows : The limb having been brought into perfect position, a slender steel bar attached above to an encircling thoracic band, and terminating just above the knee in a thigh band, was closely applied along the posterior aspect of the joint, after the manner of Thomas. The long traction brace was then applied as usual. Thus flexion was prevented, additional fixation assured, combined with effective protection. By dividing the function of the two braces, the posterior or miniature Thomas brace could be made very light and comfortable; it, however, was not to be used as a lever to correct deformity. This should first be overcome by traction in bed or otherwise. He believed this division of labor to be more practicable than the addition of perineal bands and traction to the ordinary Thomas brace, as suggested by Lovett and De Pass.

Dr. Judson commended the use of one apparatus, the hip splint, to protect the joint, and another, the antero-posterior lever, if apparatus is necessary for this purpose, to oppose flexion. In general, it is better not to attempt too many things by one and the same apparatus. He thought the antero-posterior lever, for combating flexion and maintaining fixation, was the essential element of the Thomas splint.

Dr. Shaffer said that where supplementary apparatus is employed to limit the motion of the dorso-lumbar spine and the motion on the acetabulum, unnecessary traumatism was inflicted upon the acetabulum. He had studied the subject quite closely, and, in his opinion, this motion of the dorso-lumbar spine is one of the greatest aids in the treatment of this condition. It was better to treat flexion by recumbency and rest until the flexion is overcome, than to apply an apparatus which antagonizes the very strong action of the flexor muscles.

Dr. Whitman said he recognized the force of what Dr. Shaffer had said about the flexibility of the lumbar spine, but he was inclined to think that the motion of the diseased joint which the simple traction brace permitted, and the deformity which it did not prevent, were more important considerations than the theoretical objection which Dr. Shaffer had presented. This fixation apparatus was applied before there was any flexion, and in the case presented there was no spasm of any of the muscles.

### DOES SCOLIOSIS EVER GIVE RISE TO PRESSURE MYELITIS?

Dr. H. W. Berg read a paper with the above title.

#### DISCUSSION.

Dr. H. R. Sayre thought there was no doubt that the differences in mammary development observed in cases of rotary lateral curvature were the result of trophic change, but the cause of this disturbance was still uncertain. In advanced cases, he had been inclined to attribute this disturbance to pressure on the nerves at their exit from the bony canal. Pathological specimens showed not only a narrowing of the bony canal, but also large exostoses at the points where the vertebræ join; it was quite possible that these might project inward as well as outward.

The case described in the paper had at one time been under his care, and he had considered it as closely resembling disseminated sclerosis, although it was not typical of any diseased condition with which he was familiar. Dr. Spitzka had held the same position. The case had been diagnosticated as lateral sclerosis by one neurologist, and as hysteria by another eminent neurologist, who had employed hypnotism upon the patient, though unsuccessfully. She had been referred to the speaker with the idea that there was some pressure on the cord at about the tenth dorsal vertebra, which might possibly be relieved by a surgical operation. He had been unable, however, to detect any mass pressing upon the cord, and from the effects of momentary suspension, he did not think this method of treatment would prove beneficial. He did not associate the cord lesion with the lateral curvature. The trophic changes were probably due to disturbances of nutrition external to the cord.

Dr. Shaffer considered that the author's case of lateral curvature differed only in degree from almost every case of this condition. It was rare to find lateral curvature without an exaggerated tendon reflex, a non-deforming club-foot, or various trophic changes, and the latter occur in incipient cases, before there can be any pressure on the cord. Girls suffering from lateral curvature are usually peculiarly nervous, and oftentimes seem to assume the responsibilities of their entire families. This is the direct result of the central nervous lesion, one which pertains more to the psychical condition than to the spinal cord condition. Our clinical studies drive us by analogy to look in the motor tract of the brain for the cause of the condition.

Dr. KETCH looked upon the trophic changes as an element in the etiology of lateral curvature, rather than the result of this condition. It was probable that at a very early period in life there was a disturbance of the nervous system, most probably of the brain, which produced the lateral curvature. Boys having lateral curvature, show atrophy of the limbs, but the general nervousness is not so marked. For example, he had at present under observation, a robust boy, fifteen years old, with lateral curvature, who was supernaturally strong and supernaturally slow and apathetic. He thought it highly improbable that pressure myelitis ever occurred in these cases.

Dr. L. W. HUBBARD could not understand how the paraplegia of Pott's disease could be said to be due to cord pressure from change of position, as clinically it seemed to bear no relation to the amount of curvature, or to the situation of the lesion, and it was present when there was no curvature ; and, moreover, recovery took place without any change in the curve of the spine. He saw nothing in the case reported analogous to the myelitis of Pott's disease.

Dr. JUDSON would eliminate muscular contraction as a factor in the causation of lateral curvature, believing that rotation and the curvatures, primary and secondary, are only the mechanical result of muscular failure to sustain the weight of the trunk. He would welcome with extreme pleasure any advance in our exact knowledge of the etiology of lateral curvature.

Dr. V. P. GIBNEY had never seen pressure myelitis in an uncomplicated case of rotary lateral curvature.

The CHAIRMAN agreed with Dr. Hubbard, that the analogy of the case under discussion to the myelitis of Pott's disease was not very strong, as, according to the view advanced by Dr. Hoffa at the last meeting of the American Orthopedic Association, and generally accepted by those present, the paraplegia is due to the pressure of inflammatory products. Personally, he had never seen a case of lateral curvature complicated by paraplegia or symptoms of lateral sclerosis. Last Fall he had had a case of very moderate curvature with a very peculiar ataxic gait, but a careful examination excluded organic disease of the spinal cord, and it was decided to be a case of functional nervous disturbance, possibly produced by masturbation. It seemed strange that such a mild case as the one described in the paper should produce such marked nervous symptoms, while the much more severe cases so often seen have no analogous symptoms. He looked upon the cord lesion as merely a coincidence.

Dr. Berg, in closing the discussion, said that he thought the diagnosis of disseminated sclerosis very improbable, and this diagnosis had probably been made because a primary sclerosis of the cord is such a rare condition that whenever a neurologist sees a spastic paralysis in an adult, and can find no cerebral symptoms, or symptoms of pressure upon the cord, he makes a diagnosis of disseminated sclerosis. S. Weir Mitchell had given it as his opinion, that the case was one of primary lateral sclerosis. There was no doubt as to the sclerosis and the lateral curvature, the only doubt is as to the connection between the lateral curvature ; and the sclerosis. Pott's paraplegia is caused by a variety of conditions, but he believed that in nearly seventy-five per cent. of the cases, the paraplegia was due to pressure resulting from flexion of the cord at the angle of the curve. He had no doubt that hundreds of cases had been seen where the lateral curvature had been considered the result of paralysis, where it was really the cause.

### FEMORAL ADDUCTION, ABDUCTION AND FLEXION.

Dr. Judson presented a convenient method of observing the degrees of motion in cured and convalescing cases of hip disease. The subject was illustrated by boards on which dolls were fixed, the center of motion at the hip in each case being surrounded by a graduated arc with the degrees numbered from zero, in the natural position of supine recumbency, with a slight lordosis, up to the widest limit of normal motion. In practice, the region of motion is first to be found, and then the extent to which it may be pushed, without disturbing the natural and symmetrical position of the lumbar vertebræ and the iliac spines, is to be noted on the goniometer. The degrees of motion in flexion and laterally may thus be readily recorded.

The presence of considerable motion warrants a serious effort to reduce whatever deformity may exist. He cited two cases in which the patients being considered cured, relief had been sought for the deformity. Enough motion was found to encourage hope, and good results were recorded in a few months in each case after the application of a hip splint, and later, a simple ischiatic crutch, and the return of the patient by instruction and drill to the natural rhythm of walking. The improvement was readily measured in degrees from time to time, and the deformity was almost completely reduced.

### A NEW METHOD OF MAKING PLASTER CASTS OF THE THORAX IN CASES OF ROTARY LATERAL CURVATURE.

Dr. Mary Putnam Jacobi exhibited a series of models which she had prepared by an original method. It had been suggested to her by observations made with the crytometer upon the condition of the thorax after empyema. An outline of the thorax at the desired level is first taken with a crytometer, which is an instrument consisting of two soft strips of lead united by a hinge, which is placed over the vertebral column, and the lead strips closely applied to the chest walls. The lead is next placed upon a slab of marble, where it serves as a sort of shallow frame, into which the plaster of Paris cream is poured, and allowed to set. This gives practically a thin plaster cast representing a section of the thorax.

She called attention to the ease with which the diagonal diameter could be obtained, and also to the way in which these casts brought out small degrees of curvature.

Discussion on the papers of Drs. Judson and Jacobi postponed.

